# Global Burden of Premenstrual Syndrome and Uterine Fibroids in Women of Reproductive Age (1990–2023) and Projections to 2050: An Analysis of the Global Burden of Disease Study 2023

**DOI:** 10.1002/hsr2.72602

**Published:** 2026-06-28

**Authors:** Ding Xiaoli, Yang Qiang, Wang Jun, Nawsherwan, Li Shuang

**Affiliations:** ^1^ Department of Gynecology Taixing People's Hospital Taizhou China; ^2^ Xiamen Cardiovascular Hospital of Xiamen University, School of Medicine Fujian Branch of National Clinical Research Center for Cardiovascular Diseases Xiamen China

**Keywords:** epidemiology, gynecological diseases, premenstrual syndrome, socio‐demographic index, uterine fibroids

## Abstract

**Background:**

Premenstrual syndrome (PMS) and uterine fibroids (UF) are major gynecological diseases that markedly affect the reproductive health of women of reproductive age (WRA). This study aimed to provide updated global estimates of these disorders among WRA (15–49 years) between 1990 and 2023 and to forecast future trends.

**Methods:**

Data from the GBD 2023 were used to assess the temporal trend and future projections of PMS and UF among WRA. Estimated annual percent change (EAPC), ARIMA model, and Pearson correlation analysis were used to assess temporal trends (1990–2023), forecast projections (2024–2050), and examine the association of socio‐demographic index (SDI) with major gynecological disorders, respectively.

**Results:**

In 2023, the global incidence was approximately 233,405,777 cases of PMS and 10,017,494 cases of UF. Between 1990 and 2023, the age‐standardized prevalence rates of PMS and UF among WRA increased, with EAPC of 0.06% (95% CI: 0.04, 0.07) and 0.58% (95% CI: 0.53, 0.62), and the corresponding age‐standardized disability‐adjusted life years (DALYs) rates also increased, with EAPC of 0.05% (95% CI: 0.03, 0.07) and 1.21% (95% CI: 1.01, 1.42), respectively. Projections to 2050 indicate a slight decline in PMS prevalence rates but a mild increase in DALY rates, whereas both the prevalence and DALY rates of UF are expected to rise substantially. The burden and temporal patterns of PMS and UF varied markedly across countries, regions, and SDI levels. The prevalence rates of PMS (r = 0.351) and UF (r = 0.381) showed significant positive correlations with SDI (*p* < 0.0001). The PMS burden was highest among women aged 15–30 years, while women aged 35–49 years showed a greater burden of UF.

**Conclusion:**

The global burden of PMS and UF has increased over the last 34 years, underscoring the need for targeted public health strategies to improve reproductive health in women.

**Abbreviations**
ASIR: age‐standardized incidence rate
ASPR: age‐standardized prevalence rate
ASR: age‐standardized rate
DALYs: disability‐adjusted life‐years
EAPCs: Estimated annual percentage changes
GBD: Global Burden of Disease Study
PMS: premenstrual syndrome
SDI: Sociodemographic Index
UF: uterine fibroids
WRA: women of reproductive age

## 1 Introduction

Gynecological diseases ranked second globally for new cases among non‐communicable diseases affecting women of reproductive age (WRA) and first in terms of disability‐adjusted life years (DALYs). Despite considerable advancements in global health over the past three decades, progress in reducing the burden of these diseases has been significantly slower compared to many other non‐communicable diseases [1]. The global burden of gynecological diseases, such as Premenstrual Syndrome (PMS) and Uterine Fibroids (UF), poses a significant yet frequently overlooked challenge to women's health, especially among those of reproductive age. WRA, defined as those between 15 and 49 years old, are especially vulnerable to these health conditions [2]. For instance, PMS, characterized by a range of emotional and physical symptoms that occur in the luteal phase of the menstrual cycle, affects 50% to 80% of WRA; reported prevalence varies widely by diagnostic criteria and study methods [3]. Symptoms can include mood disturbances, anxiety, irritability, and physical discomfort such as bloating and breast tenderness, which can disrupt daily functioning and overall quality of life [4]. Similarly, UF are non‐cancerous growths that affect up to 70–80% of women by age 50, based on histological (lifetime) prevalence, often resulting in complications like heavy menstrual bleeding, pelvic pain, and fertility challenges [5].

The economic impact of these gynecological conditions is considerable, leading to heightened healthcare costs and reduced productivity, as women may miss work or require medical interventions [6]. Despite their significant health implications, most existing studies primarily focus on individual conditions, specific populations, or aggregate data across all age groups, with few addressing gynecological diseases specifically within the reproductive‐age demographic. Moreover, methodological inconsistencies and the often mild or asymptomatic nature of early‐stage gynecological diseases frequently result in their underreporting in routine health statistics [7]. Furthermore, the changing landscape of risk factors, such as delayed childbearing, increasing rates of metabolic syndrome, and greater exposure to environmental endocrine disruptors, highlights the urgent need for a systematic assessment of trends over time [8, 9, 10]. The burden of gynecological diseases varies widely across regions and socio‐demographic groups, making it crucial to investigate geographical disparities and shifts in prevalence [2]. As global attention on women's health grows, there is an immediate need for accurate and comprehensive data on the estimates of gynecological diseases [11].

To date, different studies have explored the burden of PMS and UF [12, 13, 14, 15, 16, 17, 18, 19]; However, to date, few studies have provided a detailed investigation of these gynecological diseases. A significant gap remains in the global understanding of PMS and UF, particularly regarding long‐term trends in their incidence, prevalence, and DALYs. A comprehensive analysis of these metrics is essential to fully quantify their burden on women's health. Notably, no study has yet utilized the latest Global Burden of Disease (GBD) 2023 data to determine these temporal trends or to forecast future projections for PMS and UF. The GBD 2023 dataset presents a critical opportunity to address this gap by applying standardized case definitions and, where applicable, leveraging diagnostic criteria from leading bodies such as the American College of Obstetricians and Gynecologists (ACOG) [20]. This study aims to fill a critical evidence gap by conducting a detailed systematic analysis of the global PMS and UF burden among WRA by using data from the GBD 2023 study. The detailed systematic analysis will quantify long‐term trends, highlight regional disparities, and forecast future burden, providing essential data to inform public health strategies. Ultimately, this work underscores the necessity of integrating gynecological health into broader women's health agendas to guide policymakers, healthcare providers, and researchers in developing targeted interventions to improve health outcomes for women worldwide.

## 2 Materials and Methods

### 2.1 Overview and Methodological Details

The Global Burden of Disease (GBD) study is considered one of the most extensive and methodical efforts in global epidemiological research and is managed by the Institute for Health Metrics and Evaluation (IHME) at the University of Washington. This pivotal initiative delivers detailed, standardized estimates of health loss caused by a wide range of diseases, injuries, and risk factors across various populations and regions [21]. The GBD framework offers a standardized platform for conducting cross‐national and regional comparative analyses of epidemiological metrics, such as incidence and mortality rates, among various populations and geographic contexts [22]. The GBD methodology measures disease burden using several key metrics: incidence, prevalence, deaths, and disability‐adjusted life years (DALYs). DALYs serve as a composite measure that combines Years of Life Lost (YLL) due to premature death with Years Lived with Disability (YLD).

In this analysis based on the GBD framework, the burden of PMS and UF was assessed using three key metrics: incidence, prevalence, and DALYs. The study focused on WRA (15–49 years), stratifying participants into 5‐year age groups ranging from 15 to 19 years up to 45–49 years. Data for PMS and UF were sourced from the Global Health Data Exchange (GHDx) and related tools. The GBD results tool for further reference can be found at GBD 2023 (http://ghdx. healthdata. org/gbd‐results‐ tool) (accessed on Oct 15, 2025) [23]. It is important to note that the analysis did not include data on race, underlying diseases, or other relevant patient characteristics, as such information was unavailable in the GBD database. Given that the GBD database is publicly accessible, this study was exempt from formal ethical review. This study was conducted in accordance with the Guidelines for Accurate and Transparent Health Estimates Reporting (GATHER) recommendations.

### 2.2 Estimation of PMS in Incidence, Prevalence, and DALYs

GBD 2023 employed four separate DisMod‐MR 2.1 models to estimate the incidence, prevalence, and disability‐adjusted life years (DALYs) of PMS. Because epidemiological studies on PMS have consistently excluded women with irregular menstruation, a post–DisMod‐MR 2.1 correction, referred to as the “pregnancy adjustment,” was applied in GBD 2023 to account for this bias.

### 2.3 Estimation of UF in Incidence, Prevalence, and DALYs

The modeling framework, along with a detailed flowchart and corresponding code for uterine fibroid (UF) estimation in the GBD 2023 study, is available at https://ghdx.healthdata.org/gbd-2023/code/nonfatal-13. The analytical process comprised three main steps [1]: systematic identification and extraction of relevant data sources [2]; data standardization and adjustment; and [3] estimation of incidence, prevalence, and disability‐adjusted life years (DALYs) using DisMod‐MR 2.1.

### 2.4 Socio‐Demographic Index (SDI)

The Socio‐Demographic Index (SDI) is a composite metric designed to reflect the socioeconomic factors that impact population health outcomes. It is calculated as the geometric mean of three normalized indices: the total fertility rate for those under 25 years, the average educational attainment for individuals aged 15 and older, and the lag‐distributed income per capita [24]. Countries and territories are categorized into five socio‐development levels based on specific SDI thresholds: low [0–0.4658), low‐middle [0.4658–0.6188), middle [0.6188–0.7120), high‐middle [0.7120–0.8103), and high [0.8103–1.0000] [25]. This classification allows for a systematic examination of the relationship between socioeconomic development and health outcomes in populations.

### 2.5 Statistical Analysis

The age‐standardized rate (ASRs) for incidence, prevalence, and DALYs of PMS and UF among WRA were estimated per 100,000 individuals, utilizing data from the GBD database, along with their respective 95% confidence intervals. The ASRs were derived using the GBD world population age standard and reported per 100,000 population. This approach allows for comparisons between different populations or the same population over time, accommodating variations in age structures.

$$\mathrm{ASR}=\frac{\sum_{i=1}^{A}a_{i}w_{i}}{\sum_{i=1}^{A}w_{i}}\times 100,000$$

Where ai and ωi represent the age‐specific rate and the number of persons (or weight) of the same age subgroup in the selected reference standard population, respectively.

To explore the global trend of PMS and UF, we calculated the estimated annual percentage change (EAPC) for the period 1990–2023. The EAPC is a frequently used statistic to describe the change in trend of disease burden. The estimated annual percent change (EAPC) and its associated 95% confidence intervals (CIs) were computed by applying regression models to analyze temporal trends throughout the entire study duration from 1990 to 2023. Fitting log‐linear regression models obtained the EAPC and its related 95% CIs to evaluate temporal trends in ASRs and numbers of incidence, prevalence, and DALYs over the same period [26]. A negative EAPC and its 95% CI's upper limit being below zero indicates a downward trend; an upward trend is suggested by a positive EAPC and its 95% CI's lower limit being above zero.

To forecast the incidence, prevalence, and DALYs of PMS and UF, we utilized the Autoregressive Integrated Moving Average (ARIMA) model. This model effectively captures patterns and seasonal fluctuations in time series data by integrating three key components: autoregression (AR), differencing (I), and moving average (MA). Its parameters, denoted as p, d, and q, represent the autoregressive order, the degree of differencing, and the moving average order, respectively. This model generates forecasts by analyzing historical data through a combination of past values (autoregressive or AR term) and the errors from previous predictions (moving average or MA term). The “integrated” (I term) aspect of ARIMA involves differencing the observed data to maintain stationarity in the time series—essentially replacing data values with the differences from their preceding values. A zero value for any of these parameters indicates its absence in the model. For example, an ARIMA (2, 1, 0) specification signifies two AR terms, one differencing step, and no MA term. To refine the AR and MA parameters, we constructed the model and calculated the autocorrelation function (ACF) and partial autocorrelation function (PACF) of the residuals. Stationarity was assessed using the Augmented Dickey‐Fuller (ADF) test, while the autocorrelation function (ACF) and partial autocorrelation function (PACF) were used to determine optimal values for p and q. The best ARIMA (p, d, q) models were selected using the Akaike information criterion (AIC) and Bayesian information criterion (BIC) to predict disease burden. The Ljung–Box Q test was used to determine whether the residuals of the selected models satisfied an independent normal distribution. Forecasts were generated for the 2024 to 2050 horizon, and 95% confidence intervals were calculated. The ARIMA model is particularly suited for short‐term and moderate‐term forecasts, where data exhibit stable autocorrelation but limited nonlinear complexity.

Moreover, the association between the SDI and ASR of PMS and UF was evaluated using the Pearson correlation coefficient (r). A two‐sided test was used, and a *p*‐value < 0.001 was considered statistically significant. The SDI is a composite measure ranging from 0.0 to 1.0. All statistical analyses and visualizations were performed using R software (version 4.5.1).

## 3 Results

### 3.1 Incidence of PMS and UF Among WRA at the Global and Regional Levels

**Figure 1:**
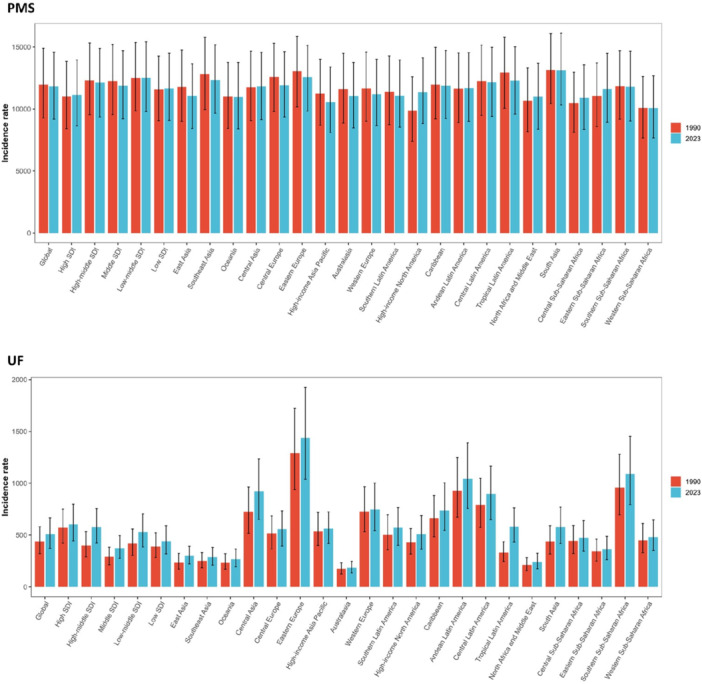
The ASIR of PMS and UF among women of reproductive age at the global and regional level between 1990 and 2023.

In 2023, the global incidence of PMS reached 233,405,777 cases, where low SDI regions recorded the highest incidence cases 50,756,907 (95% CI: 39,440,219, 62,975,558). From 1990 to 2023, the highest rise in incidence cases [EAPC = 2.75% (95% CI: 2.73, 2.77)] of PMS was also observed in the low SDI region. Among regions, high‐income North America had the highest incidence at 9,575,398 cases (95% CI: 7,450,954, 11,920,877), while the highest rise of PMS (from 1990 to 2023) was observed in Western Sub‐Saharan Africa [EAPC = 3.34% (95% CI: 3.29, 3.39)]. For UF, the global incidence cases were 10,017,494 (95% CI: 7,363,941, 13,170,590) were recorded in 2023 and high‐middle SDI countries reported the highest incidence cases 2,338,737 (95% CI: 1,718,274, 3,074,275), while the most significant rise of UF burden (from 1990 to 2023) was found in low SDI regions [EAPC = 3.16% (95% CI: 3.12, 3.2)]. Among regions, South Asia exhibited the highest incidence cases 2,899,375 (95% CI: 2,107,999, 3,874,665), and Western Sub‐Saharan Africa experienced the highest rise of UF cases [EAPC = 3.44% (95% CI: 3.37, 3.51)] from 1990 to 2023 (Table 1 and Figure 1).

**Table 1:** Incidence of PMS and UF among WRA at the global and regional levels between 1990 and 2023.

Location	Incidence of PMS				Incidence of UF			
	Incident cases (95% UI) 2023	EAPC (95%UI) 1990–2023	ASIR (per/100,000) (95% UI) 2023	EAPC (95%UI) 1990–2023	Incident cases (95% UI) 2023	EAPC (95%UI) 1990–2023	ASIR (per/100,000) (95% UI) 2023	EAPC (95%UI) 1990–2023)
Global	233405776.6 (180844087.8, 287906788.6)	1.14 (1.07, 1.21)	11831.9 (9167.4, 14594.7)	−0.05 (−0.07, −0.02)	10017494 (7363941.3, 13170590.1)	1.63 (1.55, 1.7)	507.8 (373.3, 667.6)	0.43 (0.41, 0.46)
SDI								
Low	50756907.2 (39440219.4, 62975558.1)	2.75 (2.73, 2.77)	11676.1 (9072.8, 14486.8)	0.06 (0.05, 0.07)	1912912.3 (1384873.1, 2573305.2)	3.16 (3.12, 3.2)	440.1 (318.6, 592.1)	0.46 (0.41, 0.51)
Low‐middle	39095708.6 (30566630.1, 48183951.5)	1.91 (1.84, 1.98)	12513.3 (9783.4, 15422.2)	0.03 (0.02, 0.04)	1658160.4 (1209076.9, 2195923.3)	2.74 (2.66, 2.83)	530.7 (387, 702.8)	0.84 (0.78, 0.91)
Middle	29188629.1 (22605408.2, 36143151.4)	1.33 (1.22, 1.44)	11877.8 (9198.9, 14707.8)	−0.13 (−0.16, −0.11)	919065.4 (676572.6, 1214946.1)	2.21 (2.05, 2.36)	374.1 (275.3, 494.4)	0.73 (0.69, 0.77)
High‐middle	49295555.1 (38100318.8, 60514318.4)	0.89 (0.78, 1.00)	12121.8 (9368.9, 14880.5)	−0.09 (−0.12, −0.05)	2338737.1 (1718274.6, 3074275.9)	1.98 (1.86, 2.11)	575.1 (422.5, 756)	1.0 (0.92, 1.07)
High	65068976.7 (50131609.5, 80166341.3)	0.14 (0.11, 0.18)	11352.2 (8739.2, 13993.3)	0.07 (0.03, 0.11)	3188618.8 (2342998.1, 4219899.9)	0.23 (0.06, 0.41)	556.4 (408.5, 736.9)	0.16 (0.01, 0.3)
Regions								
East Asia	35506028.3 (27020450.1, 43741536.9)	−0.32 (−0.51, −0.14)	11073.7 (8427.2, 13642.3)	−0.32 (−0.42, −0.23)	964694.2 (706735.2, 1259115.1)	0.44 (0.15, 0.74)	300.9 (220.4, 392.7)	0.44 (0.24, 0.63)
Southeast Asia	22912935.7 (17928515.8, 28193354.3)	1.12 (1.01, 1.23)	12338.3 (9654.3, 15181.7)	−0.16 (−0.18, −0.13)	530914.3 (388284.8, 709588.9)	1.62 (1.48, 1.76)	285.9 (209.1, 382.1)	0.34 (0.29, 0.38)
Oceania	396988.1 (304055.4, 498234.4)	2.68 (2.62, 2.74)	10955.9 (8391.2, 13750.1)	0.02 (0.01, 0.03)	9679.9 (7045.3, 13187.9)	2.97 (2.89, 3.05)	267.1 (194.4, 364)	0.31 (0.26, 0.35)
Central Asia	2905603.5 (2243505.2, 3582440.1)	1.06 (0.95, 1.17)	11828.7 (9133.3, 14584.1)	−0.1 (−0.14, −0.06)	226133.2 (159763.7, 303692.9)	1.63 (1.55, 1.72)	920.6 (650.4, 1236.3)	0.46 (0.34, 0.58)
Central Europe	3055219.5 (2401611.4, 3753075.1)	−0.84 (−0.92, −0.76)	11913.5 (9364.9, 14634.8)	−0.13 (−0.16, −0.1)	142951 (101186.4, 188309.5)	−0.48 (−0.58, −0.37)	557.4 (394.6, 734.3)	0.24 (0.2, 0.28)
Eastern Europe	6190028.7 (4840694.4, 7444140.3)	−0.68 (−0.81, −0.56)	12568 (9828.4, 15114.4)	−0.18 (−0.22, −0.14)	708964.1 (511373.8, 949257.2)	−0.16 (−0.26, −0.06)	1439.5 (1038.3, 1927.3)	0.34 (0.14, 0.54)
High‐income Asia Pacific	3841379.1 (2947435.1, 4870280.3)	−0.77 (−0.86, −0.69)	10566.9 (8107.9, 13397.3)	−0.17 (−0.26, −0.09)	204069.5 (151982.3, 263281.5)	−0.58 (−0.84, −0.31)	561.4 (418.1, 724.2)	0.02 (−0.21, 0.26)
Australasia	821944.1 (629132.2, 1021465.6)	0.82 (0.76, 0.88)	11062 (8467.1, 13747.2)	−0.12 (−0.13, −0.1)	13808.5 (10058.7, 18329.1)	0.93 (0.86, 1.01)	185.8 (135.4, 246.7)	0 (−0.06, 0.06)
Western Europe	10621278.6 (8202418.3, 13295875.1)	−0.18 (−0.23, −0.14)	11198.6 (8648.3, 14018.6)	−0.13 (−0.14, −0.12)	708424.4 (515010.8, 949722.1)	−0.03 (−0.16, 0.1)	746.9 (543, 1001.3)	0.02 (−0.06, 0.11)
Southern Latin America	2011323.2 (1548192.7, 2531541.1)	1.12 (1.11, 1.14)	11082.6 (8530.7, 13949)	−0.05 (−0.06, −0.04)	103497.6 (72853.9, 138711.3)	1.44 (1.34, 1.53)	570.3 (401.4, 764.3)	0.26 (0.18, 0.35)
High‐income North America	9575398.4 (7450954.1, 11920877.5)	0.81 (0.71, 0.92)	11354.7 (8835.5, 14136)	0.49 (0.41, 0.58)	428468.7 (306238.4, 580429.6)	0.98 (0.67, 1.29)	508.1 (363.1, 688.3)	0.66 (0.37, 0.95)
Caribbean	1445296.1 (1122432.9, 1791432.7)	0.78 (0.69, 0.87)	11880.6 (9226.6, 14725.9)	−0.03 (−0.05, −0.01)	89767.1 (66312.5, 122253.1)	0.92 (0.77, 1.08)	737.9 (545.1, 1004.9)	0.11 (0.01, 0.21)
Andean Latin America	2024849.3 (1556237.8, 2516547.2)	1.88 (1.79, 1.97)	11703.5 (8995, 14545.5)	0.03 (0.02, 0.05)	180405.3 (130982.6, 240458.5)	2.21 (2.14, 2.28)	1042.7 (757.1, 1389.8)	0.35 (0.32, 0.38)
Central Latin America	8486948.4 (6578726.1, 10460934.1)	1.53 (1.41, 1.65)	12144.3 (9413.7, 14968.9)	−0.01 (−0.02, 0)	627219.7 (452416.6, 814709.2)	1.85 (1.72, 1.98)	897.5 (647.4, 1165.8)	0.31 (0.27, 0.35)
Tropical Latin America	7076636.1 (5516295.1, 8633643.8)	1.01 (0.87, 1.15)	12304.5 (9591.5, 15011.7)	−0.12 (−0.15, −0.09)	333989.5 (247673.2, 438431.7)	2.89 (2.68, 3.09)	580.7 (430.6, 762.3)	1.74 (1.64, 1.85)
North Africa and Middle East	17639617.7 (13433702.8, 21978314.7)	2.28 (2.12, 2.44)	10987.8 (8367.9, 13690.4)	0.08 (0.06, 0.11)	387365.4 (282117.5, 523080.4)	2.62 (2.48, 2.75)	241.3 (175.7, 325.8)	0.41 (0.37, 0.46)
South Asia	66085113.4 (51960660.7, 81114803.1)	2.16 (2.08, 2.24)	13124.9 (10319.7, 16109.9)	0.02 (0.01, 0.04)	2899375.1 (2107999.4, 3874665.3)	3.19 (3.07, 3.31)	575.8 (418.7, 769.5)	1.03 (0.93, 1.14)
Central Sub‐Saharan Africa	3763108.6 (2885452.5, 4689567.8)	3.3 (3.26, 3.34)	10891.7 (8351.5, 13573.2)	0.13 (0.09, 0.17)	163590.1 (118998.2, 220715.9)	3.33 (3.31, 3.35)	473.5 (344.4, 638.8)	0.16 (0.14, 0.18)
Eastern Sub‐Saharan Africa	13204760.3 (10161920.4, 16461788.2)	3.17 (3.15, 3.2)	11609.3 (8934.1, 14472.9)	0.2 (0.18, 0.21)	411670.4 (297957.8, 554915.2)	3.2 (3.17, 3.24)	361.9 (262, 487.9)	0.23 (0.2, 0.26)
Southern Sub‐Saharan Africa	2890211.5 (2209064.8, 3591528.2)	1.62 (1.52, 1.71)	11809.1 (9026, 14674.6)	−0.02 (−0.04, −0.01)	266963.2 (194542.6, 355547.3)	2.04 (1.94, 2.14)	1090.8 (794.9, 1452.7)	0.39 (0.34, 0.45)
Western Sub‐Saharan Africa	12951107.3 (9859989.7, 16280786.7)	3.34 (3.29, 3.39)	10080.8 (7674.7, 12672.5)	0.04 (−0.02, 0.11)	615542.9 (449957.5, 828460.3)	3.44 (3.37, 3.51)	479.1 (350.2, 644.9)	0.14 (0.09, 0.19)

Abbreviations: ASIR, age‐standardized incidence rate; CI, confidence interval; EAPC, estimated annual percentage change; SDI, socio‐demographic index; UI, uncertainty interval.

In 2023, the global age‐standardized incidence rate (ASIR) for PMS was 11,831.9 per 100,000 people (95% CI: 9,167.4 to 14,594.7). Low‐middle SDI regions reported the highest ASIR at 12,513.3 per 100,000 (95% CI: 9,783.4 to 15,422.2), while high‐income SDI regions exhibited the highest rise in rate of PMS [EAPC 0.07% (95% CI: 0.03, 0.10) from 1990 to 2023. In regions, South Asia had the highest ASIR for PMS 13,124.9 (95% CI: 10,319.7, 16,109.9), and the highest rise in burden of PMS from 1990 to 2023 was observed in Central Sub‐Saharan Africa [EAPC = 0.13% (95% CI: 0.09, 0.17). For UF, the global ASIR was 507.8 (95% CI: 373.3, 667.6), and high SDI countries demonstrated the highest ASIR for UF 604.8 (95% CI: 445.3, 800.1), while the highest rise (from 1990 to 2023) in burden was noted in high‐middle‐income SDI regions [EAPC = 1.00% (95% CI: 0.92, 1.07)]. Among regions, Eastern Europe exhibited the highest ASIR for UF 1,439.5 (95% CI: 1,038.3, 1,927.3), and Tropical Latin America recorded the highest rise among regions [EAPC = 1.74% (95% CI: 1.64, 1.85) from 1990 to 2023 (Table 1 and Figure 1).

### 3.2 Prevalence of PMS and UF Among WRA at the Global and Regional Levels

In 2023, the global prevalence of PMS reached 897,068,642.5 cases. High‐middle SDI regions accounted for the highest prevalence cases, 191,783,330.6 (95% CI: 153,256,066.5, 230,572,600.4), and the highest rise in PMS burden (from 1990 to 2023) was recorded in low SDI regions [EAPC = 2.8% (95% CI: 2.78, 2.81)]. Among regions, Southeast Asia reported the highest prevalence of cases, 88,685,368.4 (95% CI: 71,652,674.9, 106,042,589.8), while the highest rise (from 1990 to 2023) in cases of PMS was observed in Western Sub‐Saharan Africa [EAPC = 3.38% (95% CI: 3.33, 3.42)]. For UF, the global prevalence cases were 87,280,767.03 (95% CI: 63,559,097.77, 116,244,612.4) in 2023. In 2023, middle SDI countries reported the highest prevalence cases 8,062,073.04(95% CI: 5,899,025.16, 10,715,604.12), while the highest rise (from 1990 to 2023) in cases was noted in low SDI regions [EAPC = 3.24% (95% CI: 3.20, 3.28)]. Among regions, Southern Latin America exhibited the highest prevalence of UF at 941,388.22 cases (95% CI: 668,289.23, 1,288,781.47) in 2023, and Western Sub‐Saharan Africa had the highest rise (from 1990 to 2023) in cases of UF [EAPC = 3.56% (95% CI: 3.49, 3.63) (Table S1 and Figure S1).

In 2023, the global age‐standardized prevalence rate (ASPR) for PMS was 45,474.6 per 100,000 (95% CI: 36,553.5 to 54,675.7) and low‐middle SDI regions recorded the highest ASPR 47,303.8 (95% CI: 38,653.3, 56,086.5), while high‐income SDI regions exhibited the highest rise in burden of PMS [EAPC = 0.12% (95% CI: 0.09, 0.15)] from 1990 to 2023. In regions, Eastern Europe reported the highest ASPR for PMS 50,289.3 (95% CI: 40,189.5, 60,046.2) in 2023, and the highest rise (from 1990 to 2023) in rate was observed in high‐income North America [EAPC = 0.50% (95% CI: 0.41, 0.58). For UF, the global ASPR reached to 4,424.47 (95% CI: 3,221.96, 5,892.72) and High SDI countries had the highest ASPR at 5,894.97 (95% CI: 4,265, 7,897.6) in 2023, while the highest rise (from 1990 to 2023) was noted in high‐middle‐income SDI regions [EAPC = 1.32% (95% CI: 1.26, 1.38)]. Additionally, Southern Sub‐Saharan Africa exhibited the highest ASPR for UF 9,315.78 (95% CI: 6,718.65, 12,574.71) in 2023, and Tropical Latin America recorded the highest rise (from 1990 to 2023) in UF rate [EAPC = 1.78% (95% CI: 1.69, 1.87)] among the regions (Table S1 and Figure S1).

### 3.3 DALYs of PMS and UF Among WRA at the Global and Regional Levels

In 2023, the global DALYs for PMS reached approximately 7,545,304 cases, and high middle SDI regions accounted for the largest share of DALYs cases, 1,612,654.52 (95% CI: 1,009,186.94, 2,405,025.23), while the highest rise in cases from 1990 to 2023 was observed in low SDI areas [EAPC = 2.81% (95% CI: 2.80, 2.83). In 2023, Central Asia reported the highest DALYs cases 94,404.19 (95% CI: 58,472.63, 141,520.92), and the highest rise in cases from 1990 to 2023 of PMS was noted in Western Sub‐Saharan Africa [EAPC = 3.39% (95% CI: 3.35, 3.43)] among regions. In 2023, the global DALYs of UF reached approximately 158,989.1 cases, and low SDI countries recorded the highest DALYs cases, at 60,211.1 (95% CI: 29,381.8, 111,278.1), and the greatest rise in DALYS cases from 1990 to 2023 was found in low SDI areas [EAPC = 3.34 (95% CI: 3.12, 3.57)]. In regions, South Asia exhibited the highest DALYs cases of UF 77,405.3 (95% CI: 41,680.15, 144,586.17) in 2023, while Central Sub‐Saharan Africa had the highest rise in burden of UF [EAPC = 4.43% (95% CI: 4.05, 4.81) (Table S2 and Figure S2) from 1990 to 2023.

In 2023, the global age‐standardized disability‐adjusted life year (ASDR) rate for PMS reached 382.4 per 100,000 population approximately and low middle SDI regions reported the highest ASDR 396.9 (95% CI: 244.4, 592.87), while low SDI regions showed the highest rise in ASDR of PMS [EAPC = 0.13% (95% CI: 0.12, 0.13)] from 1990 to 2023. In regions, Eastern Europe recorded the highest ASDR of PMS 423.2 (95% CI: 264.1, 630.4), and the highest rise from 1990 to 2023 in ASDR was found in Eastern Sub‐Saharan Africa [EAPC = 0.5% (95% CI: 0.41, 0.58)]. For UF, the global ASDR reached 8.1 per 100,000 in 2023. Low SDI countries exhibited the highest ASDR 13.8 (95% CI: 6.7, 25.6), while the greatest rise (from 1990 to 2023) in ASDR of UF was observed in high middle‐income SDI regions [EAPC = 1.64% (95% CI: 1.46, 1.83). In 2023, South Asia had the highest ASDR for UF 15.3 (95% CI: 8.2, 28.7), and Tropical Latin America recorded the highest rise from 1990 to 2023 in ASDR of UF of [EAPC = 2.12% (95% CI: 2.00, 2.25) among regions (Table S2 and Figure S2).

### 3.4 Age‐Specific Global Burden of PMS and UF

**Figure 2:**
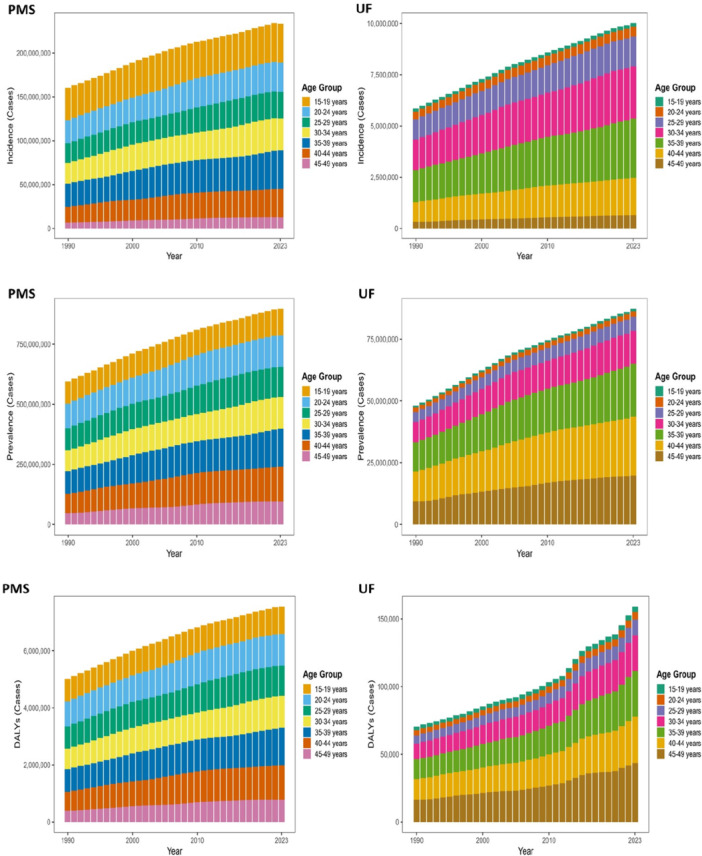
Global incidence, prevalence, and DALYs cases of PMS and UF for different age groups between 1990 and 2023.

Results are showing that global incidence, prevalence, and DALYs cases of PMS are high and in increasing manner among early age groups (15–19 years, 20–24 years, 25–29 years). On the other hand, incidence, prevalence, and DALYs cases of UF are high in older age groups (35–39 years, 40–44 years, 45–49 years) (Figure 2).

### 3.5 Burden and Trend of PMS and UF at the Country Level

**Figure 3:**
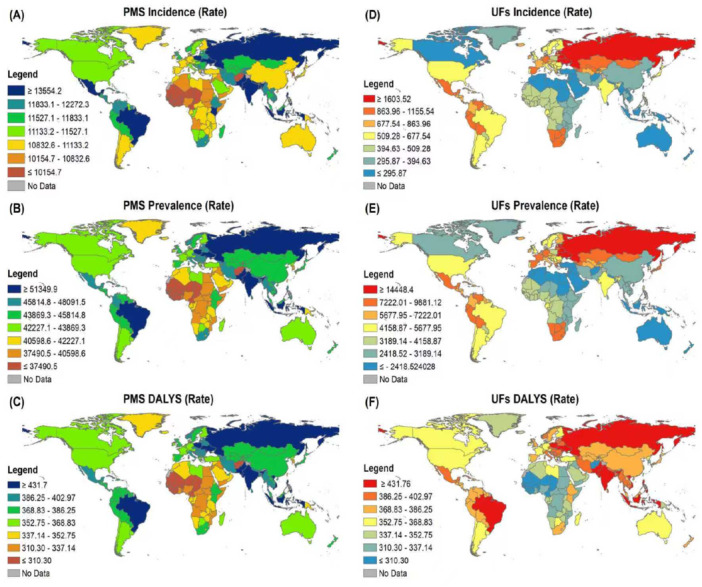
Age‐standardized rate (ASR) of incidence, prevalence, and DALYs of PMS and UF among WRA at the country level in 2023. Panels A–C show the ASRs of PMS, including incidence (A), prevalence (B), and DALYs (C). Panels D–F show the ASRs of UFs, including incidence (D), prevalence (E), and DALYs (F). Countries are colored according to the corresponding ASR categories, and grey indicates countries or territories with no available data.

**Figure 4:**
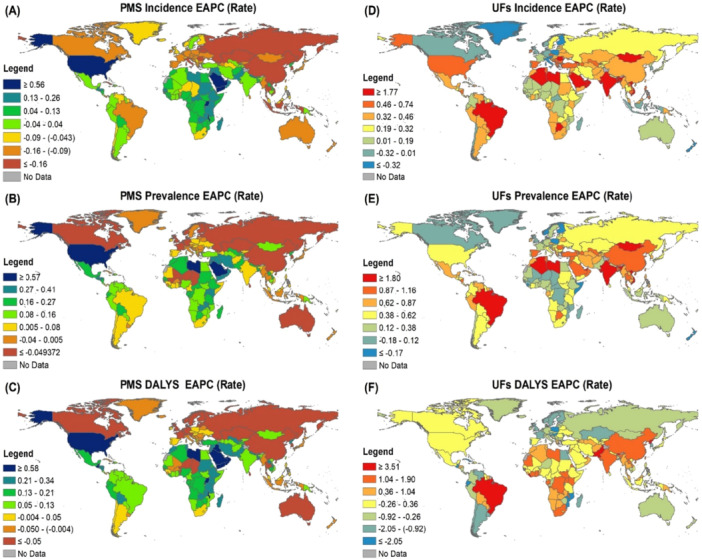
Estimated Annual percentage of change (EPAC) in rate of incidence, prevalence, and DALYS of PMS and UF among WRA at the country level from 1990 to 2023. Panels A–C present the EAPC in PMS‐related incidence (A), prevalence (B), and DALYs (C). Panels D–F present the EAPC in UF‐related incidence (D), prevalence (E), and DALYs (F). Countries are colored according to the corresponding EAPC categories, and grey indicates countries or territories with no available data.

Our findings reveal that, in 2023, the highest ASIR of PMS was observed in Pakistan (13554.26 per 100,000 people), followed by India (13138.19 per 100,000 people), while the highest ASPR for PMS was reported in Ukraine (51349.98 per 100,000 people), followed by Pakistan (50558.10 per 100,000 people). In terms of PMS‐related DALYS rate (ASDR), again, Ukraine had the highest rate, 431.76 per 100,000 people, followed by Pakistan, 424.9942297 per 100,000 people. For UF, the highest ASIR (1603.520648 per 100,000 people) and ASPR (14448.42 per 100,000 people) were observed in Latvia, followed by Russia [(ASIR: 1450.65 per 100,000 people) and (ASPR: 12946.54435 per 100,000 people)]. However, the UF‐related DALYs rate (ASDR) showed a different pattern, with Saint Kitts and Nevis reporting the highest rate (30.20 per 100,000 people), followed by Eritrea (27.28 per 100,000 people) (Figure 3). From 1990 to 2023, the USA experienced the most significant rise in both ASIR [EAPC = 0.57% (0.46, 0.67)] and ASDR [EAPC = 0.58% (0.49, 0.68)] for PMS, while the UAE showed the greatest increase in ASPR [EAPC = 0.58% (0.48, 0.68)] for PMS. In contrast, Brazil saw the highest rise in ASIR [EAPC = 1.78% (1.67, 1.89)] and ASPR [EAPC = 1.81% (1.72, 1.89) for UF, and Fiji had the most significant increase in ASDR [EAPC = 3.52% (2.92, 4.11)] for UF (Figure 4) In terms of new cases, India reported the highest number of incidence, prevalence, and DALYs cases for both PMS and UF (Figure S3). Meanwhile, between 1990 and 2023, Qatar exhibited the most significant increase in new cases of incidence, prevalence, and DALYs for both PMS and UF (Figure S4).

### 3.6 Association Between the Burden of PMS, UF, and the SDI

**Figure 5:**
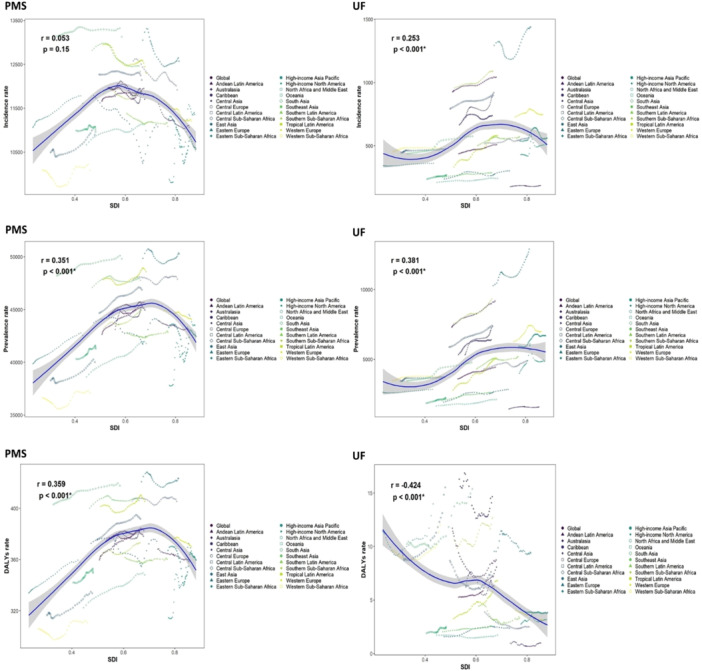
Association between the incidence, prevalence, and DALY rates (per 100,000 population) of PMS and UF with the SDI among WRA, 1990–2023.

The SDI serves as an indicator of healthcare quality and accessibility across nations. To investigate the relationship between SDI and the burden of PMS and UF, we conducted a Pearson correlation analysis (Figure 5). The expected values based on SDI and age‐standardized rates (ASR) for all locations are represented by the solid black line. Each point reflects the observed ASR for individual countries in 2023. Countries situated above the black line experience a higher‐than‐expected burden, while those below the line have a lower‐than‐expected burden. Positive correlations were found between PMS prevalence (r = 0.351, *p* < 0.001), and DALY rates (r = 0.359, *p* < 0.001) with SDI, indicating that a country with higher SDI is associated with a greater burden of PMS. In terms of UF, positive correlations were observed between SDI and UF incidence (r = 0.253, *p* < 0.001) and prevalence (r = 0.381, *p* < 0.001). However, a negative correlation was identified between DALY rates for UF and SDI (r = −0.424, *p* < 0.001). This indicates that while higher SDI is associated with increased incidence and prevalence of UF, it corresponds to a reduction in the burden of disability‐adjusted life years.

### 3.7 Projection of PMS and UF (2024–2050)

**Figure 6:**
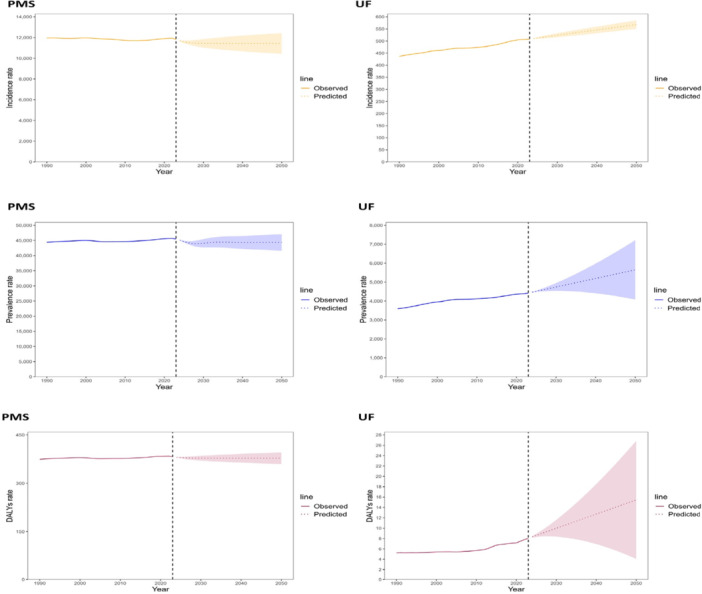
Global projections of incidence, prevalence, and DALYs rate (per 100,000 population) of PMS UF among WRA between 1990 and 2050.

The ARIMA model was utilized to forecast global projections of incidence, prevalence, and DALYs rates (per 100,000 population) and number of cases for PMS and UF among WRA from 2024 to 2050. The analysis suggests that by 2050, similar trends of PMS incidence, prevalence, and DALYs rates are likely to persist. However, a decline in PMS incidence and prevalence cases is expected (Figures 4 and S3). Conversely, the DALYs cases for PMS are projected to see a slight increase by 2050. In contrast, the burden of UF, including both rates and cases, is projected to increase by the year 2050 (Figures 6 and Figure S5).

## 4 Discussion

To the best of our knowledge, this study provides the most comprehensive and up‐to‐date assessment of the global, regional, and national burden of PMS and UF among women of reproductive age (15–49 years). Using the GBD 2023 data spanning 1990–2023 and incorporating projections to 2050, we found that both gynecological diseases impose a substantial and growing global health burden. Our analysis reveals marked heterogeneity across age groups, geographic regions, countries, and SDI levels, underscoring significant inequities in disease distribution and outcomes. These findings highlight critical gaps in prevention, diagnosis, and management, particularly in low‐ and middle‐SDI settings, and provide robust evidence to inform targeted public health policies, resource allocation, and future research aimed at improving women's reproductive health worldwide.

### 4.1 Global and Regional Burden of PMS and UF

In 2023, PMS showed increasing incidence, prevalence, and DALYs, consistent with prior evidence of its substantial impact on women's health. Global incidence exceeded 23 million cases, markedly higher than in earlier years [27]. Low and low‐middle SDI regions reported the highest PMS incidence and ASIR, likely reflecting greater vulnerability among lower socioeconomic groups due to limited healthcare access and higher stress levels [14]. Regionally, North America reported the highest number of PMS incident cases, whereas South Asia had the highest ASIR, likely reflecting cultural and environmental influences; similar patterns have been reported in previous studies showing higher PMS incidence in South Asian populations [28]. The marked rise in PMS prevalence over the past 33 years is largely driven by population growth, with globalization and urbanization contributing through higher‐calorie diets and increased adiposity that may worsen PMS symptoms [29]. Our study found low‐middle SDI regions reported the highest ASPR, aligning with previous studies indicating that countries with lower SDI often have the highest ASPR [16]. Regionally, Southeast Asia reports the highest prevalence, which aligns with previous findings indicating higher PMS rates in Asian countries compared to European countries [30]. The highest increase in PMS prevalence was observed in Western Sub‐Saharan Africa, indicating a rapid rise in PMS cases, a finding supported by previous studies [31]. Limited healthcare access, gender inequity, and socioeconomic challenges exacerbate PMS and hinder accurate reporting. Women in these regions also face a triple burden of neglected tropical diseases, non‐communicable diseases, and reproductive health conditions, further worsening health outcomes [15, 32]. This pattern aligns with the previous study (2023), which links socioeconomic stressors to more severe PMS symptoms [33]. Moreover, an increasing trend in DALYs for PMS was observed, contrasting with a previous study that reported an opposite trend [28]. High‐middle SDI regions contribute significantly to DALYs.

In contrast, low‐middle SDI regions report high DALY rates (ASDR), consistent with previous studies [16], reflecting challenges such as limited healthcare access and high infectious disease prevalence. Central Asia shows high DALYs, aligning with prior research [34]. On the other hand, Eastern Europe, with notable ASDR, may face socioeconomic and post‐Soviet health system challenges, suggesting that comprehensive health reforms could reduce mortality rates [35]. Our study shows marked regional variation in PMS burden, with the highest levels in low to low‐middle SDI regions. Limited healthcare, poor education, economic challenges, and widespread misconceptions about menstruation reduce support for women and worsen PMS impacts, leading to a higher incidence [36]. Targeted health programs and improved education are essential to reduce these disparities.

In 2023, UF incidence continued to rise globally. High‐middle SDI countries had the greatest number of cases. At the same time, low SDI regions experienced the fastest increase, consistent with prior evidence showing a growing UF burden in middle to low SDI quintiles [37]. Rapid economic development likely drives this trend by promoting unhealthy dietary shifts, physical inactivity, and increased exposure to endocrine‐disrupting chemicals—including plasticizers, dioxins, and phthalates—thereby elevating UF risk [38, 39, 40]. Regionally, South Asia exhibited the highest number of UF cases, and Western Sub‐Saharan Africa experienced the highest rise. These results are consistent with studies suggesting that women originating from South Asian, African, and Middle Eastern subcontinents are more prone to fibroids [37, 41]. In addition, the global ASIR for UF was recorded at 507.8 per 100,000 people, with notably higher rates observed in high SDI countries. This trend contrasts sharply with previous studies, which often indicated that low SDI countries had higher rates of ASIR for UF [42]. Eastern Europe has the highest ASIR of UF, while Tropical Latin America shows the fastest increase over the last three decades. Reportedly, lifestyle and dietary changes linked to rapid economic development, along with genetic, racial, and gynecological factors, obesity, poor diet, and pelvic inflammatory disease, likely contribute to this trend [43]. Moreover, Improved reproductive health awareness and healthcare access may also increase reported incidence as more women seek care [44]. In 2023, an overall increasing trend in the prevalence of UF was observed, aligning with findings from previous studies [14]. Middle SDI countries reported the highest prevalence of UF, while low SDI regions showed the greatest increase in cases, consistent with previous studies [13].

In contrast, high SDI countries had the highest ASPR, and the largest rise in prevalence occurred in high‐middle SDI regions, differing from earlier findings [37]. Regionally, Southern Latin America had the highest UF prevalence, Western Sub‐Saharan Africa had the largest increase in prevalence cases, Southern Sub‐Saharan Africa had the highest ASPR, and Tropical Latin America had the fastest growth in ASPR. These findings align with prior studies showing rising UF prevalence, particularly in Africa, Asia, North America, and South America [14]. In 2023, an increasing trend in DALYs for UF was observed, which is in contrast to a previous study where a decreasing trend was observed in 2019 [37]. Low SDI countries had the highest DALY burden, and DALYs cases increased most in low SDI regions, while ASDR rose fastest in high‐middle SDI regions; prior studies similarly report a high UF burden in middle to low SDI quintiles [13]. South Asia recorded the highest burden of DALYs of UF, which is in line with a previous study where the same trend was observed [12]. Similarly, Central Sub‐Saharan Africa and Tropical Latin America showed the largest increases in UF DALY cases and ASDR, respectively. Consistent with prior evidence, African and African‐descended women, particularly African‐American women, have a two to threefold higher risk of UF than other populations, as demonstrated by ultrasound screening, clinical, and pathological studies [45, 46, 47]. Future UF prevention and treatment may face major challenges due to regional disparities. As the burden increasingly shifts to low‐ and middle‐SDI countries, limited healthcare resources, inadequate screening, suboptimal treatment, and financial barriers impede early diagnosis and care. Although overall incidence has declined in high‐income countries, high‐risk groups persist, and lifestyle factors—such as poor diet, physical inactivity, pollution, and delayed childbirth—further complicate control efforts. Addressing socioeconomic disparities, particularly in middle‐ and low‐SDI regions, is essential to improve UF management [48, 49, 50].

Moreover, our study revealed rising incidence, prevalence, and DALYs of PMS among women aged 15–29 years. It is likely driven by hormonal fluctuations, unhealthy lifestyles, high stress, and coexisting mental health conditions, as reported in previous studies [51]. In contrast, the incidence, prevalence, and DALYs of UF are substantially higher in older reproductive‐age women, particularly those aged 35–49 years. This pattern reflects hormone‐dependent fibroid growth driven by estrogen and progesterone during the late reproductive years. Consequently, women aged 30–45 should be prioritized for public health interventions, including expanded access to uterine‐sparing therapies, individualized hormonal treatments, and integration of fibroid screening into routine gynecologic care to enable earlier detection and reduce surgical burden [52].

### 4.2 Burden and Trend of PMS and UF at the Country Level

Our findings show marked national variation in PMS and UF burden. Pakistan had the highest PMS age‐standardized incidence rate (ASIR), consistent with prior studies [19], likely reflecting sociocultural and healthcare factors, including low education and limited awareness in low‐ and middle‐income settings [53]. In contrast, Ukraine exhibited the highest ASPR and DALYs rate (ASDR) of PMS, suggesting suboptimal management and resulting long‐term disability; similar patterns have been linked to underdiagnosis and limited treatment access in Eastern Europe [54]. In terms of UF, Latvia has the highest ASIR and ASPR, which aligns with a previous study [14], likely reflecting robust diagnostic capacity and effective surveillance, enabling greater detection, including asymptomatic cases, rather than a uniquely elevated disease risk [55]. Regarding the increase in rates of PMS and UF, the USA shows the highest rise across PMS incidence, prevalence, and DALYs, indicating a rapidly growing burden of PMS. This result is similar to a previous study [19]. This trend is due to greater awareness and better diagnostic capabilities, which result in more cases being pointed out increased awareness and improvements in healthcare reporting in the USA contribute to rising PMS diagnoses, along with a changing lifestyle that may exacerbate PMS symptoms [56]. For UF, Brazil leads in both incidence and prevalence, which aligns with previous findings [37], which linked higher incidence rates in Brazil to urbanization and lifestyle changes, which may contribute to earlier diagnoses and a higher reporting rate.

India has the highest total number of PMS and UF cases, reflecting its large population and consistent with the greater chronic disease burden seen in densely populated regions [57]. The rising prevalence in India has been linked to urbanization, dietary changes, and delayed childbearing [58]. In contrast, Qatar shows the highest increase of PMS and UF incidence, prevalence, and DALYs, indicating a rapidly increasing burden likely driven by improved healthcare access, lifestyle changes, and urbanization, trends also reported across the Gulf region [59].

### 4.3 Projection of PMS and UF (2024–2050)

Projection analyses suggested that by 2050, current PMS trends will persist, with declines in incidence and prevalence likely due to improved awareness, earlier recognition, better healthcare access, and public health initiatives promoting healthy lifestyles and stress management [60]. However, a modest increase in DALYs is projected, indicating a continued substantial burden. This may reflect greater symptom severity, mental health comorbidities, population aging with more women experiencing premenopausal symptoms, and socioeconomic disparities limiting access to effective care [61, 62]. These findings highlight the need for sustained public health efforts focused on education, equitable healthcare access, and targeted PMS interventions. Conversely, while PMS DALYs are projected to increase slightly by 2050, the burden of UF is expected to rise substantially in both rates and cases. This increase may be associated with population aging, higher obesity prevalence, unhealthy lifestyles, improved detection with greater awareness, and persistent socioeconomic and healthcare disparities that limit access to effective prevention and treatment [50]. These contrasting trends underscore the need for targeted strategies to address both PMS and UF and to ensure adequate support for women throughout their reproductive years.

### 4.4 Association Between the Burden of PMS, UF, and the SDI

The positive association between PMS incidence, prevalence, and DALY rates with the SDI suggests that higher‐SDI countries report a greater PMS burden, likely due to better diagnostic recognition, increased health‐seeking behavior, and greater awareness of menstrual health rather than a true increase in disease occurrence. Improved access to reproductive and mental health services and reduced stigma in these settings enable more accurate documentation of functional impairment. In contrast, underdiagnosis and sociocultural barriers may lead to underestimation in lower‐SDI regions [63]. In comparison, UF shows a more nuanced pattern. The positive correlations between SDI and both incidence and prevalence suggest that advanced imaging availability (e.g., ultrasound, MRI) and routine gynecological screening in high‐SDI countries increase detection rates. However, the negative correlation between DALYs and SDI highlights the critical role of healthcare quality. In high‐SDI regions, fibroids are more likely to be diagnosed early, monitored effectively, and treated with minimally invasive or fertility‐sparing procedures. These factors reduce symptom severity, prevent complications, and ultimately lower disability despite higher detection rates. Conversely, in low‐SDI countries, limited access to screening and treatment often results in late diagnosis and unmanaged symptoms, contributing to a greater disability burden even when incidence and prevalence are underestimated [64]. Together, these patterns underscore how diagnostic capacity shapes the apparent burden, whereas treatment availability determines the disability impact, revealing important inequities in women's health across global development levels.

### 4.5 Limitations

This study has several limitations. Reliance on GBD data limited information on race, comorbidities, and other key patient characteristics, potentially masking demographic influences on PMS and UF burden. Stratification by 5‐year age groups may obscure within‐group socioeconomic and lifestyle differences, and geographic variations in healthcare access and quality were not fully captured. Heterogeneity in study designs complicates comparisons, while ARIMA‐based projections rely on historical trends that may not reflect future changes in healthcare or social behaviors. The effectiveness of current interventions was not assessed, limiting actionable insights. Although exempt from ethical review due to the use of public data, the absence of informed consent raises ethical considerations. These limitations highlight the need for more detailed, context‐specific research on PMS and UF.

### 4.6 Implications

This study highlights the need for targeted public health strategies to improve PMS and UF management. Key actions include enhancing awareness and education to promote early recognition and care‐seeking, expanding access to diagnostics and treatment, particularly in low‐ and middle‐income regions, and integrating menstrual health into broader health education to address misconceptions and support healthy lifestyles. Future research should generate more comprehensive demographic and clinical data to guide tailored interventions and policies, alongside prioritizing funding to evaluate the effectiveness of existing treatments and optimize resource allocation.

## 5 Conclusion

In conclusion, this study demonstrates that the global burden of PMS and UF among women of reproductive age has increased substantially from 1990 to 2023, with pronounced disparities across age groups, regions, countries, and SDI levels. PMS predominantly affects younger women, while UF imposes a greater burden on women in later reproductive years. Low‐ and middle‐SDI regions bear a disproportionate share of disability, reflecting persistent inequities in healthcare access, diagnosis, and management. Projections to 2050 suggest a modest decline in PMS incidence and prevalence but a continued rise in related disability, alongside a marked increase in UF burden. These findings highlight the urgent need for age‐ and region‐specific public health strategies, improved access to reproductive and mental health services, and sustained investment in prevention, early detection, and effective management to reduce the long‐term impact of PMS and UF on women's health globally.

## Author Contributions

**Ding Xiaoli:** conceptualization, investigation, writing – original draft, methodology, visualization, writing – review and editing, formal analysis, project administration, data curation. **Yang Qiang:** conceptualization, investigation, writing – original draft, methodology, writing – review and editing, visualization, software, data curation. **Wang Jun:** conceptualization, methodology, writing – review and editing, formal analysis, data curation. **Nawsherwan:** conceptualization, writing – review and editing, visualization, methodology, supervision, investigation, validation. **Li Shuang:** conceptualization, methodology, validation, writing – review and editing, project administration, supervision, resources.

## Funding

The authors have nothing to report.

## Ethics Statement

All authors have read and approved the final version of the manuscript. Nawsherwan had full **a**ccess to all of the data in this study and takes complete responsibility for the integrity of the data and the accuracy of the data analysis.

## Consent

The authors have nothing to report.

## Conflicts of Interest

The authors declare no conflicts of interest.

## Transparency Statement

Nawsherwan affirms that this manuscript is an honest, accurate, and transparent account of the study being reported; that no important aspects of the study have been omitted; and that any discrepancies from the study as planned (and, if relevant, registered) have been explained.

## Supporting information

Supporting File 1

Supporting File 2

## Data Availability

The data that supports the findings of this study are available in the supplementary material of this article.
