# Supplementary material for: Global Burden of Premenstrual Syndrome and Uterine Fibroids in Women of Reproductive Age (1990–2023) and Projections to 2050: An Analysis of the Global Burden of Disease Study 2023
Source: Health Sci Rep. 2026 Jun 28;9(7):e72602. doi: 10.1002/hsr2.72602 (PMC13311307; doi:10.1002/hsr2.72602)
Supplement: Supplementary file 1 — Supporting File 1 [file HSR2-9-e72602-s002.docx]

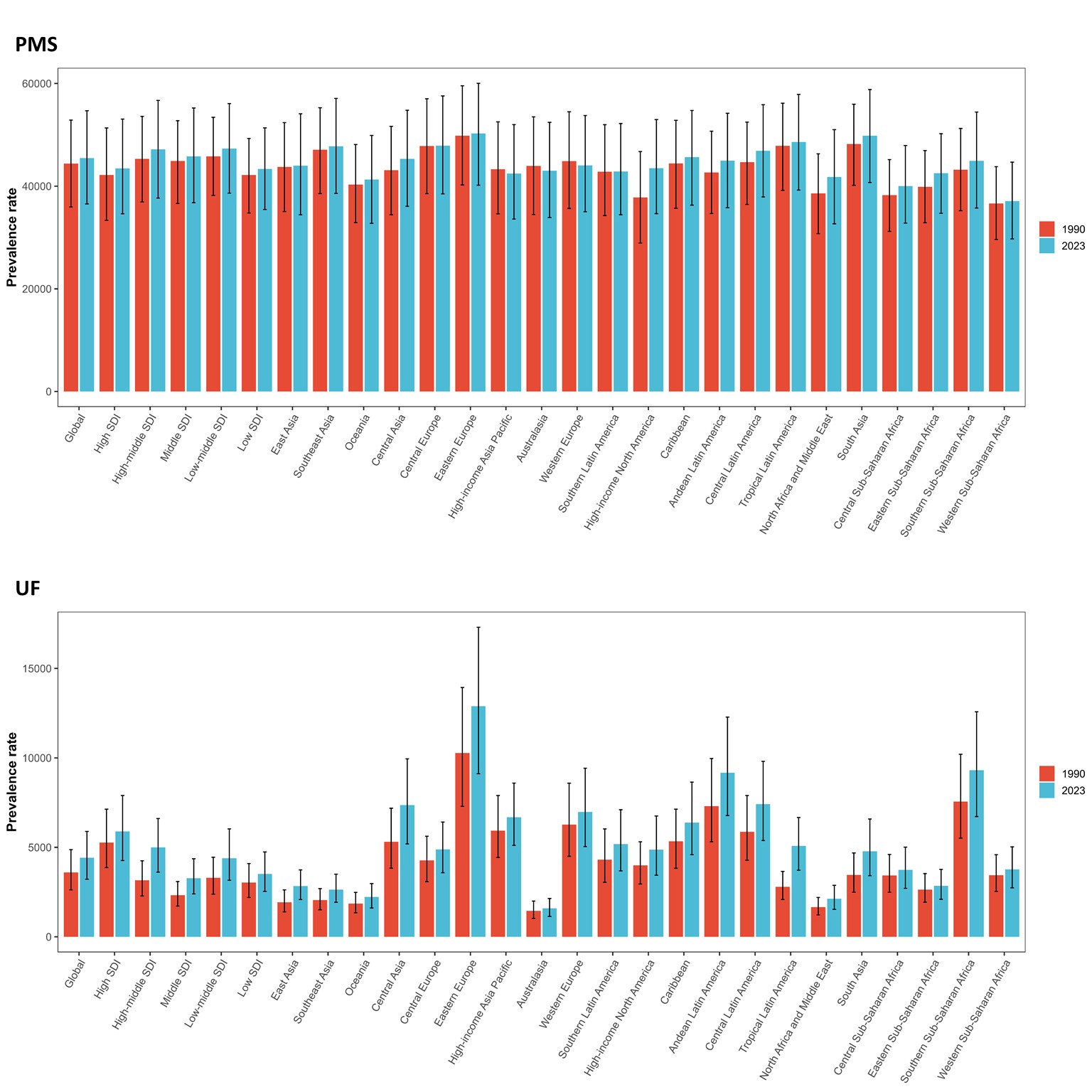


**Figure S1:** The ASR of prevalence of PMS and UF among WRA at the global and regional level between 1990 and 2023**.**


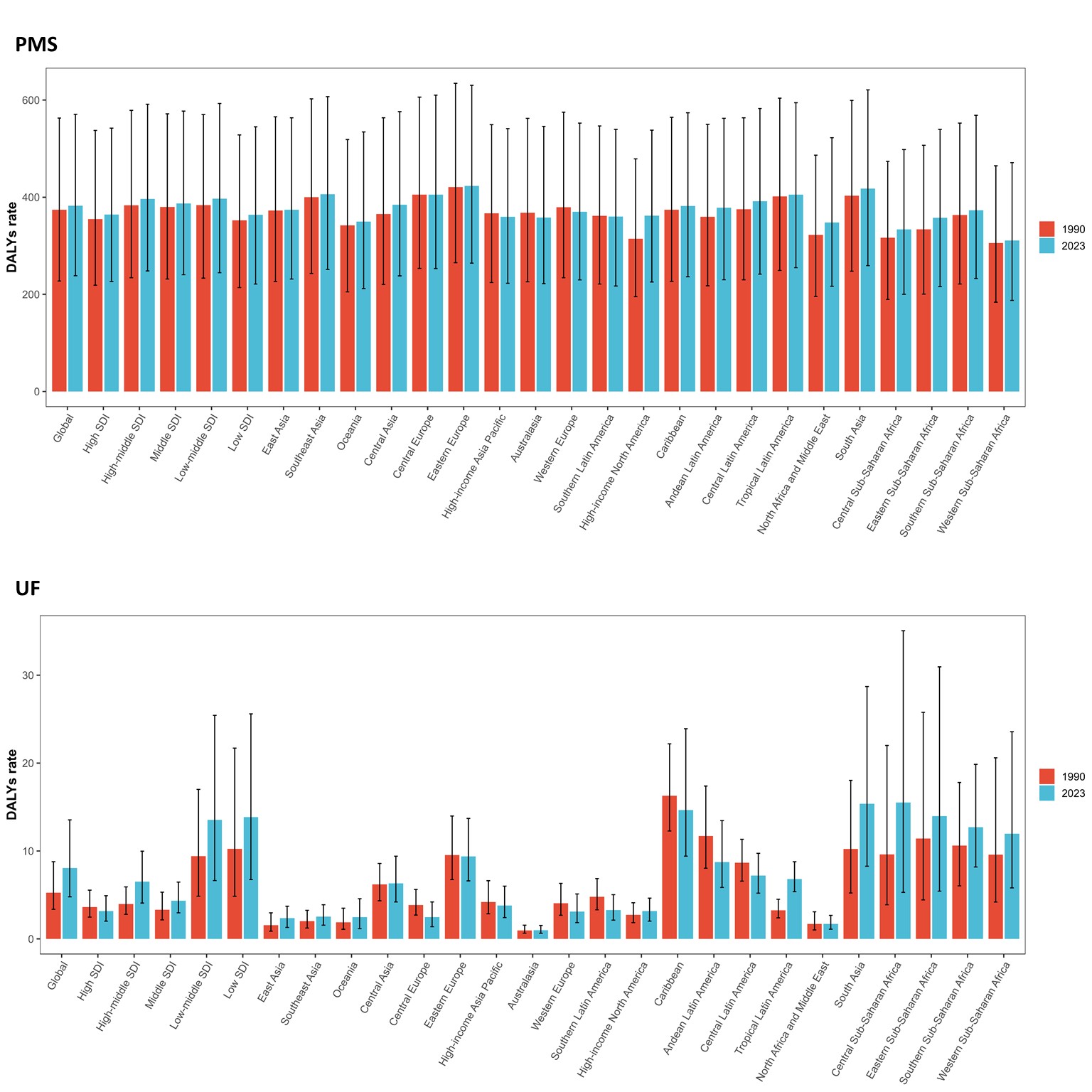


**Figure S2:** The ASR of DALYs of PMS and UF among WRA at the global and regional level between 1990 and 2023**.**


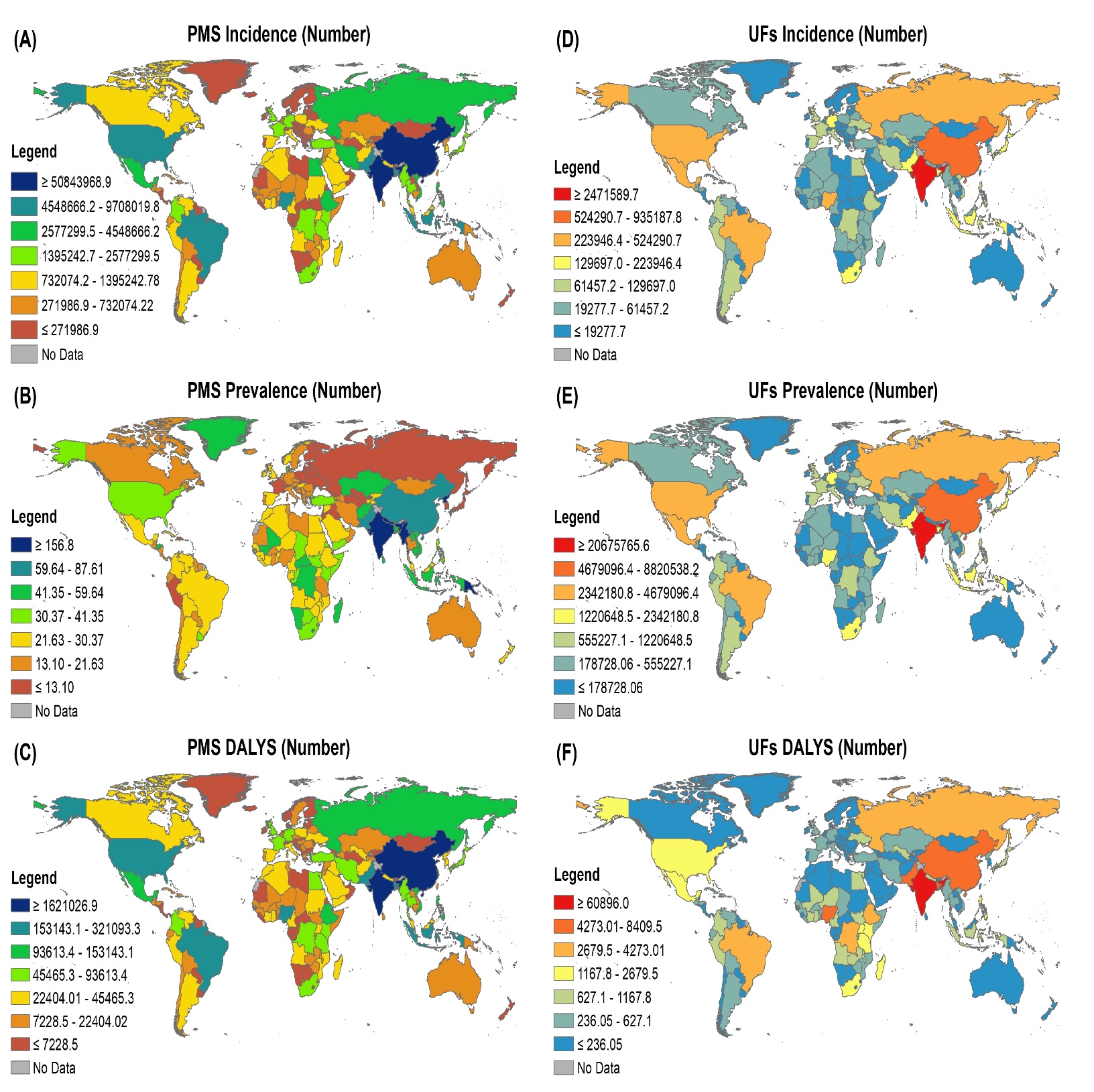


**Figure S3:** Cases of Incidence, Prevalence and DALYS of PMS and UFs among WRA at country level in 2023.


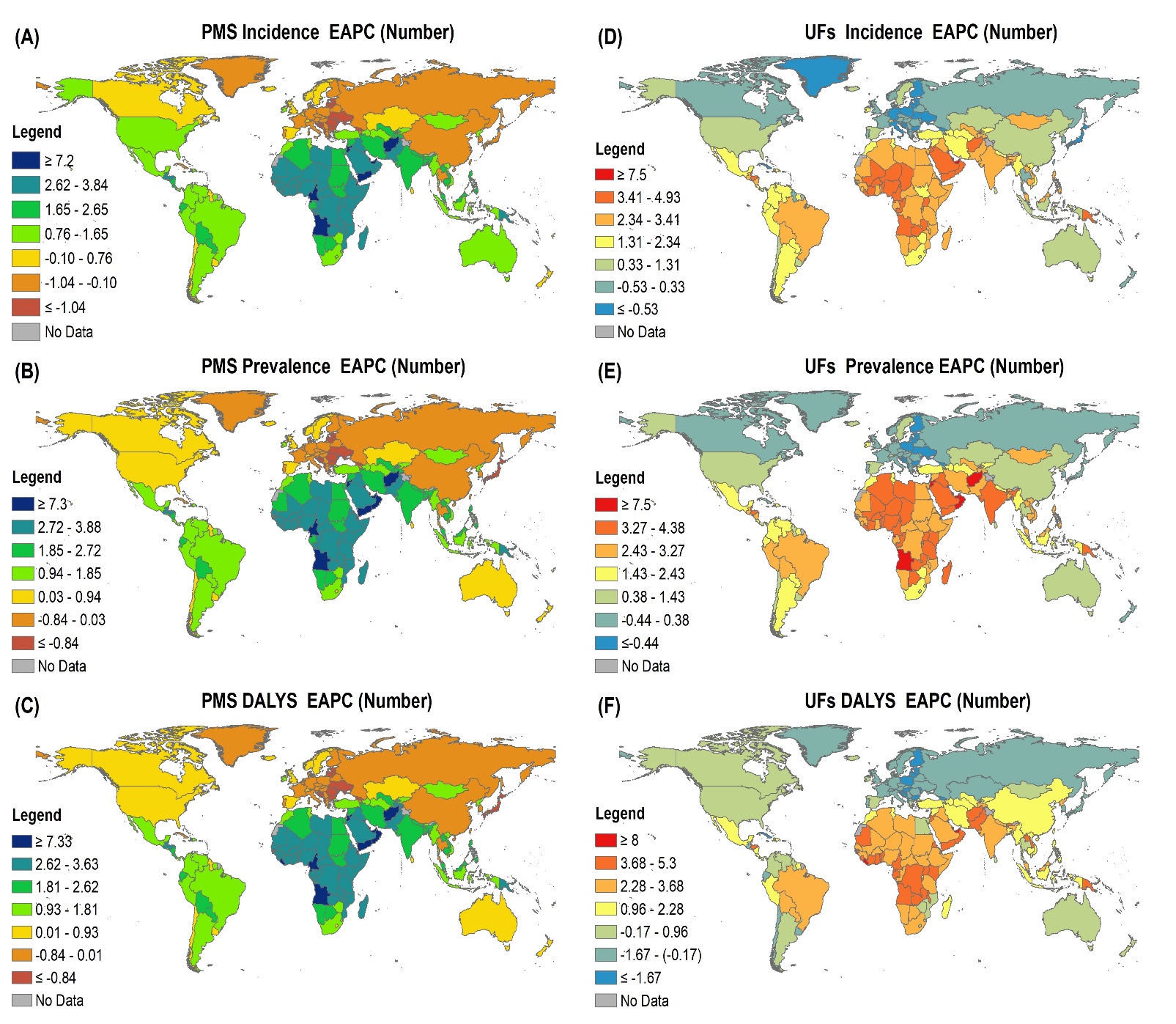


**Figure S4:** Estimated Annual percentage of change (EPAC) in cases of Incidence, Prevalence and DALYS of PMS and UFs among women of reproductive age at country level from 1990-2023.


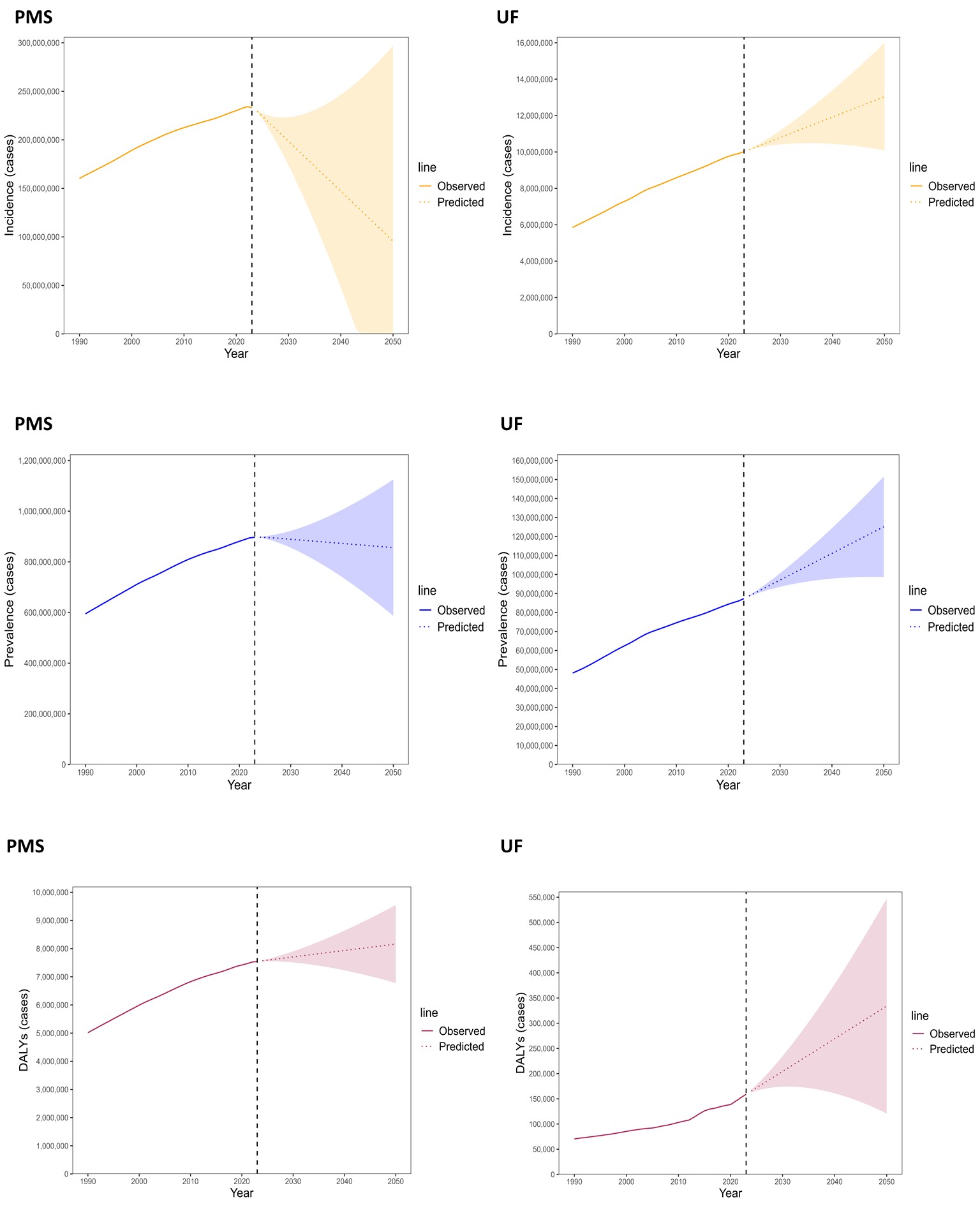


**Figure S5:** Global projections of incidence, prevalence, and DALYs cases of premenstrual syndrome (PMS) and uterine fibroids (UF) among women of reproductive age between 1990 and 2050.
