# Supplementary material for: Global Burden of Premenstrual Syndrome and Uterine Fibroids in Women of Reproductive Age (1990–2023) and Projections to 2050: An Analysis of the Global Burden of Disease Study 2023
Source: Health Sci Rep. 2026 Jun 28;9(7):e72602. doi: 10.1002/hsr2.72602 (PMC13311307; doi:10.1002/hsr2.72602)
Supplement: Supplementary file 2 — Supporting File 2 [file HSR2-9-e72602-s001.docx]

**Table S1:** Prevalence of PMS and UF among WRA at the global and regional levels between 1990 and 2023

| Location | Prevalence of PMS | | | | Prevalence of UF | | | |
| --- | --- | --- | --- | --- | --- | --- | --- | --- |
|  | Prevalence cases  (95% UI)  2023 | EAPC (95%UI) in cases  1990–2023 | ASR of prevalence  (95% UI)  2023 | EAPC (95%UI) in rate  1990–2023 | Prevalence cases  (95% UI)  2023 | EAPC (95%UI) in cases  1990–2023 | ASR of prevalence  (95% UI)  2023 | EAPC (95%UI) in rate  1990–2023 |
| Global | 897068642.5 (721084525.1, 1078578144) | 1.25 (1.17, 1.32) | 45474.6 (36553.5, 54675.7) | 0.06 (0.04, 0.07) | 87280767.1 (63559097.7, 116244612.4) | 1.77 (1.65, 1.89) | 4424.4  (3221.9, 5892.7) | 0.58 (0.53, 0.62) |
| SDI |  |  |  |  |  |  |  |  |
| Low | 188482008.5 (154078056.5, 223259618.9) | 2.8 (2.78, 2.81) | 43358.2 (35444, 51358.4) | 0.10 (0.1, 0.11) | 15257016.2  (11040007.4, 20607918.7) | 3.24 (3.2, 3.28) | 3509.7 (2539.6, 4740.6) | 0.54 (0.49, 0.59) |
| Low-middle | 147792610.8 (120765631.6, 175232646.3) | 2 (1.93, 2.06) | 47303.8 (38653.3, 56086.5) | 0.11 (0.11, 0.12) | 13715341.1 (9886448.2, 18838014.5) | 2.9 (2.81, 2.98) | 4389.8 (3164.3, 6029.4) | 0.99 (0.93, 1.06) |
| Middle | 112564165.9 (90380707, 135741423.5) | 1.49 (1.38, 1.6) | 45806 (36778.9, 55237.6) | 0.03 (0.01, 0.04) | 8062073.1 (5899025.1, 10715604.1) | 2.52 (2.34, 2.7) | 3280.7 (2400.5, 4360.5) | 1.04 (0.98, 1.1) |
| High-middle | 191783330.6 (153256066.5, 230572600.4) | 1.06 (0.96, 1.17) | 47159.7 (37685.8, 56698) | 0.08 (0.06, 0.11) | 20316563.1 (14719602.8, 26871107.4) | 2.31 (2.17, 2.45) | 4995.8 (3619.5, 6607.6) | 1.32 (1.26, 1.38) |
| High | 256446526.6 (201997268.5, 312000396.2) | 0.2 (0.15, 0.24) | 44743.1 (35217.9, 54461.4) | 0.12 (0.09, 0.15) | 29929773.4 (21860601.0, 39684274.2) | 0.37 (0.15, 0.59) | 5224.3 (3812.7, 6930.7) | 0.29 (0.11, 0.48) |
| Regions |  |  |  |  |  |  |  |  |
| East Asia | 141044535.6 (110370081.7, 173501740.4) | -0.08 (-0.26, 0.09) | 43989.4 (34422.6, 54112.3) | -0.09 (-0.15, -0.02) | 9097337.6 (6677542.5, 11988066.2) | 0.95 (0.6, 1.3) | 2837.3 (2082.6, 3738.8) | 0.94 (0.75, 1.14) |
| Southeast Asia | 88685368.4 (71652674.9, 106042589.8) | 1.28 (1.17, 1.39) | 47755.8 (38584, 57102.5) | 0 (-0.01, 0.02) | 4891512.8 (3594189.3, 6497260.3) | 3.34 (3.22, 3.46) | 2634.1 (1935.4, 3498.6) | 0.71 (0.65, 0.78) |
| Oceania | 1495801.5 (1187146.9, 1807859.7) | 2.76 (2.7, 2.82) | 41280.5 (32762.4, 49892.6) | 0.1 (0.09, 0.11) | 80905.8 (58436.1, 107757.1) | 3.17 (3.08, 3.25) | 2232.8 (1612.7, 2973.8) | 0.5 (0.46, 0.54) |
|  |  |  |  |  |  |  |  |  |
| Central Asia | 11129450 (8860610.2, 13458155.2) | 1.23 (1.12, 1.34) | 45308 (36071.6, 54788.2) | 0.07 (0.03, 0.1) | 1808523.2 (1275199.4, 2444620.3) | 1.97 (1.8, 2.14) | 7362.5 (5191.3, 9952.1) | 0.8 (0.7, 0.9) |
| Central Europe | 12282926.9 (9870742.9, 14762876) | -0.7 (-0.79, -0.61) | 47896.1 (38490, 57566.4) | 0.01 (-0.01, 0.03) | 1253835.9 (918162.9, 1645889.4) | -0.47 (-0.63, -0.32) | 4889.2 (3580.2, 6417.9) | 0.24 (0.15, 0.33) |
| Eastern Europe | 24768532.3 (19794178.1, 29574040) | -0.54 (-0.68, -0.41) | 50289.3 (40189.5, 60046.2) | -0.04 (-0.07, -0.01) | 6347129.3 (4488208.8, 8522691.7) | -0.02 (-0.18, 0.14) | 12887.1 (9112.7, 17304.2) | 0.48 (0.37, 0.59) |
| High-income Asia Pacific | 15430819 (12210259.3, 18897030.1) | -0.66 (-0.76, -0.57) | 42447.4 (33588.2, 51982.3) | -0.06 (-0.16, 0.04) | 2429899.7 (1859162.5, 3122312.1) | -0.16 (-0.31, -0.01) | 6684.2 (5114.2, 8588.9) | 0.45 (0.34, 0.56) |
| Australasia | 3196364.8 (2518157.2, 3895061) | 0.88 (0.83, 0.93) | 43017.7 (33890.2, 52421) | -0.05 (-0.06, -0.05) | 117977.2 (84685.4, 159145.4) | 1.04 (0.96, 1.11) | 1587.7 (1139.7, 2141.8) | 0.1 (0.01, 0.19) |
| Western Europe | 41754789.6 (33224424.1, 50975181.9) | -0.11 (-0.17, -0.05) | 44024.4 (35030.4, 53746) | -0.06 (-0.06, -0.05) | 6617140.1 (4780881.4, 8934578.8) | 0.26 (0.06, 0.45) | 6976.8 (5040.7, 9420.2) | 0.31 (0.17, 0.46) |
| Southern Latin America | 7775255.6 (6251400.1, 9475075) | 1.19 (1.17, 1.2) | 42842.4 (34445.8, 52208.5) | 0.02 (0.01, 0.03) | 941388.2 (668289.2, 1288781.4) | 1.55 (1.43, 1.67) | 5187.1 (3682.3, 7101.3) | 0.38 (0.27, 0.49) |
| High-income North America | 36676179.1 (29214780.6, 44667340.9) | 0.82 (0.72, 0.92) | 43491.4 (34643.5, 52967.5) | 0.5 (0.41, 0.58) | 4106723.4 (2907610.1, 5696623.3) | 0.71 (0.33, 1.09) | 4869.8 (3447.9, 6755.1) | 0.38 (0.04, 0.73) |
| Caribbean | 5553478.5 (4417226.7, 6658671.8) | 0.88 (0.78, 0.97) | 45650.5 (36310.3, 54735.4) | 0.06 (0.05, 0.08) | 777078.3 (558568.2, 1051558.8) | 1.26 (1.07, 1.44) | 6387.7 (4591.5, 8643.9) | 0.44 (0.34, 0.55) |
| Andean Latin America | 7779418.7 (6192434.1, 9380079.8) | 2.03 (1.94, 2.11) | 44964.7 (35792, 54216.5) | 0.18 (0.17, 0.19) | 1586362.6 (1172290.1, 2125169.7) | 2.54 (2.47, 2.62) | 9169.1 (6775.7, 12283.4) | 0.68 (0.66, 0.71) |
| Central Latin America | 32758352.1 (26479886.7, 39036171.7) | 1.7 (1.58, 1.82) | 46875.1 (37891.1, 55858.2) | 0.16 (0.14, 0.17) | 5182226.1 (3764632.1, 6852648.5) | 2.22 (2.09, 2.36) | 7415.4 (5386.9, 9805.69) | 0.67 (0.64, 0.71) |
|  |  |  |  |  |  |  |  |  |
| Tropical Latin America | 27947047.1 (22563827, 33278476.6) | 1.2 (1.06, 1.34) | 48592.9 (39232.8, 57862.9) | 0.07 (0.05, 0.1) | 2921193.3 (2140983.9, 3836751.5) | 2.92 (2.73, 3.12) | 5079.2 (3722.6, 6671.1) | 1.78 (1.69, 1.87) |
| North Africa and Middle East | 67069282.7 (52458675.3, 81868977.1) | 2.43 (2.28, 2.58) | 41777.9 (32676.8, 50996.7) | 0.23 (0.21, 0.24) | 3407934.2 (2458088.2, 4616225.6) | 2.93 (2.82, 3.03) | 2122.8 (1531.1, 2875.4) | 0.71 (0.64, 0.78) |
| South Asia | 250848130.8 (204990801.6, 296222406.2) | 2.25 (2.18, 2.32) | 49819.8 (40712.3, 58831.4) | 0.11 (0.11, 0.12) | 24065532.1 (17205982.6, 33172438.7) | 3.34 (3.22, 3.46) | 4779.5 (3417.2, 6588.2) | 1.18 (1.07, 1.29) |
| Central Sub-Saharan Africa | 13828063.9 (11332808.3, 16556605.3) | 3.31 (3.28, 3.35) | 40023.1 (32801, 47920.4) | 0.14 (0.11, 0.18) | 1291041.7 (935563.4, 1732729.3) | 3.38 (3.34, 3.43) | 3736.7 (2707.8, 5015.1) | 0.21 (0.17, 0.25) |
| Eastern Sub-Saharan Africa | 48386180.5 (39488792.1, 57131548.5) | 3.2 (3.18, 3.23) | 42540.1 (34717.7, 50228.8) | 0.23 (0.21, 0.24) | 3239702.5 (2383429.8, 4291611.1) | 3.25 (3.2, 3.31) | 2848.2 (2095.4, 3773.1) | 0.28 (0.23, 0.32) |
| Southern Sub-Saharan Africa | 10995172.1 (8745492, 13320361.7) | 1.74 (1.65, 1.84) | 44925 (35733, 54425.4) | 0.1 (0.08, 0.12) | 2279992.1 (1644356.7, 3077600.8) | 2.19 (2.07, 2.32) | 9315.7 (6718.6, 12574.7) | 0.54 (0.49, 0.59) |
| Western Sub-Saharan Africa | 47663493.3 (38190854.1, 57397430.7) | 3.38 (3.33, 3.42) | 37099.9 (29726.7, 44676.6) | 0.08 (0.03, 0.13) | 4837330.2 (3520917.2, 6460793.4) | 3.56 (3.49, 3.63) | 3765.2 (2740.5, 5028.9) | 0.25 (0.2, 0.31) |

**Abbreviations:** ASR, age standardized rate per/ 100,000; EAPC, estimated annual percentage change; SDI, socio-demographic index; UI, uncertainty interval; CI, confidence interval.

**Table S2:** DALYs of PMS and UF among WRA at the global and regional levels between 1990 and 2023

| Location | DALYs of PMS | | | | DALYs of UF | | | |
| --- | --- | --- | --- | --- | --- | --- | --- | --- |
|  | DALYs number  (95% UI)  2023 | EAPC (95%UI) in cases  1990–2023 | ASR of DALYs  (95% UI)  2023 | EAPC (95%UI) in rate  1990–2023 | DALYs number  (95% UI)  2023 | EAPC (95%UI) in cases  1990–2023 | ASR of DALYs  (95% UI)  2023 | EAPC (95%UI) in rate  1990–2023 |
| Global | 7545304.2 (4701853.7, 11255585.7) | 1.24 (1.17, 1.31) | 382.4  (238.3, 570.5) | 0.05 (0.03, 0.07) | 158989.1 (94485.5, 267317.3) | 2.42 (2.29, 2.55) | 8.1 (4.7, 13.5) | 1.21 (1.01, 1.42) |
| SDI |  |  |  |  |  |  |  |  |
| Low | 1580604.7 (962057.7, 2369087.9) | 2.81 (2.8, 2.83) | 363.6 (221.3, 544.9) | 0.13 (0.12, 0.13) | 60211.1 (29381.8, 111278.1) | 3.34 (3.12, 3.57) | 13.8 (6.7, 25.6) | 0.64 (0.41, 0.87) |
| Low-middle | 1240311.5 (763579.5, 1852330.4) | 2 (1.94, 2.07) | 396.9 (244.4, 592.8) | 0.12 (0.11, 0.12) | 42340.1 (20749.5, 79484.2) | 3.22 (2.9, 3.55) | 13.5 (6.6, 25.4) | 1.32 (0.95, 1.69) |
| Middle | 951224.5 (590998.7, 1418367.1) | 1.49 (1.37, 1.6) | 387.1 (240.5, 577.1) | 0.02 (0.01, 0.04) | 10674.1 (7312.1, 15897.3) | 2.25 (2.15, 2.35) | 4.3 (2.9, 6.4) | 0.78 (0.72, 0.83) |
| High-middle | 1612654.5 (1009186.9, 2405025.2) | 1.04 (0.94, 1.15) | 396.5 (248.1, 591.4) | 0.07 (0.04, 0.09) | 26469.1 (16567.4, 40571.4) | 2.64 (2.54, 2.73) | 6.5 (4.1, 9.9) | 1.64 (1.46, 1.83) |
| High | 878654.4 (545582.4, 1307258.9) | 0.19 (0.15, 0.23) | 376.9  (236.3, 560.4) | 0.11 (0.08, 0.14) | 19301.5 (13286.4, 29191.1) | -0.38 (-0.47, -0.29) | 3.3 (2.3, 5.1) | -0.46 (-0.54, -0.38) |
| Regions |  |  |  |  |  |  |  |  |
| East Asia | 1199910.6 (741914.4, 1806595.5) | -0.09 (-0.26, 0.09) | 374.2 (231.3, 563.4) | -0.09 (-0.16, -0.03) | 7563.6 (4168.7, 11943.1) | 1.59 (1.05, 2.14) | 2.3 (1.3, 3.7) | 1.59 (1.27, 1.92) |
| Southeast Asia | 754087.5 (466934.4, 1127567.6) | 1.29 (1.17, 1.4) | 406.1 (251.4, 607.1) | 0.01 (-0.01, 0.03) | 4703.1 (2917.3, 7213.1) | 1.84 (1.7, 1.98) | 2.5 (1.5, 3.8) | 0.56 (0.5, 0.62) |
| Oceania | 12671.9 (7675.4, 19358.4) | 2.76 (2.7, 2.82) | 349.7 (211.8, 534.2) | 0.1 (0.09, 0.11) | 90.02 (42.28, 165.81) | 3.54 (3.39, 3.69) | 2.4 (1.1, 4.5) | 0.86 (0.73, 0.99) |
|  |  |  |  |  |  |  |  |  |
| Central Asia | 94404.1 (58472.6, 141520.9) | 1.24 (1.12, 1.35) | 384.3 (238.1, 576.1) | 0.07 (0.04, 0.1) | 1550.8 (1034.6, 2310.2) | 0.75 (0.44, 1.06) | 6.3 (4.2, 9.4) | -0.41 (-0.75, -0.06) |
| Central Europe | 103963.2 (64904.1, 156432.4) | -0.7 (-0.79, -0.62) | 405.3 (253.1, 609.9) | 0.01 (-0.01, 0.02) | 634.4 (354.7, 1080.3) | -1.95 (-2.15, -1.75) | 2.4 (1.3, 4.2) | -1.25 (-1.53, -0.96) |
| Eastern Europe | 208448.3 (130059.6, 310486.9) | -0.54 (-0.68, -0.41) | 423.2 (264.1, 630.4) | -0.04 (-0.07, -0.02) | 4625.1 (3248.8, 6748.4) | -1 (-1.33, -0.67) | 9.3 (6.6, 13.7) | -0.5 (-0.7, -0.3) |
| High-income Asia Pacific | 130720.3 (80963.1, 196792.9) | -0.66 (-0.76, -0.57) | 359.5 (222.7, 541.3) | -0.06 (-0.16, 0.03) | 1384.1 (880.1, 2181.3) | -0.77 (-0.83, -0.71) | 3.8 (2.4, 6.1) | -0.17 (-0.23, -0.11) |
| Australasia | 26613.7 (16497.5, 40549.7) | 0.86 (0.82, 0.91) | 358.1 (222.1, 545.7) | -0.07 (-0.08, -0.07) | 72.6 (47.3, 113.6) | 0.45 (-0.17, 1.07) | 0.9 (0.6, 1.5) | -0.48 (-1.05, 0.1) |
| Western Europe | 350737.2 (217932.1, 524010.7) | -0.12 (-0.18, -0.07) | 369.8 (229.7, 552.4) | -0.07 (-0.08, -0.06) | 2940.2 (1764.5, 4857.1) | -0.85 (-0.93, -0.78) | 3.1 (1.8, 5.1) | -0.8 (-0.85, -0.75) |
| Southern Latin America | 65353.7 (39428.8, 97942.7) | 1.18 (1.16, 1.19) | 360.1 (217.2, 539.6) | 0.01 (0, 0.02) | 597.1 (386.6, 913.3) | -0.13 (-0.52, 0.27) | 3.2 (2.1, 5.1) | -1.28 (-1.69, -0.87) |
| High-income North America | 305229.3 (190199.8, 453571.4) | 0.83 (0.73, 0.94) | 361.9 (225.5, 537.8) | 0.51 (0.43, 0.6) | 2664.5 (1699.4, 3914.8) | 0.46 (0.2, 0.72) | 3.1 (2.1, 4.6) | 0.14 (-0.09, 0.37) |
| Caribbean | 46467.1 (28752.1, 69808.4) | 0.86 (0.77, 0.96) | 381.9 (236.3, 573.8) | 0.05 (0.04, 0.06) | 1782.7 (1145.8, 2908.2) | 0.13 (-0.07, 0.34) | 14.6 (9.4, 23.9) | -0.67 (-0.93, -0.41) |
| Andean Latin America | 65438.7 (39790.4, 97327.8) | 2.03 (1.94, 2.12) | 378.2 (229.9, 562.5) | 0.18 (0.16, 0.19) | 1512.3 (1012.3, 2329.3) | 0.86 (0.52, 1.21) | 8.7 (5.8, 13.4) | -0.97 (-1.38, -0.55) |
| Central Latin America | 273745.1 (168880.1, 407158.1) | 1.69 (1.57, 1.81) | 391.7 (241.6, 582.6) | 0.15 (0.13, 0.16) | 5046.7 (3642.1, 6809.1) | 1.17 (0.9, 1.45) | 7.2 (5.2, 9.7) | -0.36 (-0.73, 0.01) |
| Tropical Latin America | 233159.1 (146654.7, 341841.2) | 1.19 (1.05, 1.34) | 405.4 (255.1, 594.3) | 0.07 (0.04, 0.1) | 3913.5 (3092.9, 5046.7) | 3.27 (3.1, 3.44) | 6.8 (5.3, 8.7) | 2.12 (2, 2.25) |
| North Africa and Middle East | 558650.5 (347907.2, 838813.3) | 2.42 (2.27, 2.58) | 347.9 (216.7, 522.5) | 0.22 (0.21, 0.23) | 2748.3 (1776.6, 4312.6) | 1.93 (1.83, 2.03) | 1.7 (1.1, 2.6) | -0.26 (-0.45, -0.07) |
| South Asia | 2102956.9 (1304758.1, 3127228.4) | 2.26 (2.19, 2.33) | 417.6 (259.1, 621.1) | 0.12 (0.11, 0.13) | 77405.3 (41680.1, 144586.1) | 3.49 (3.24, 3.73) | 15.3 (8.2, 28.7) | 1.32 (1.03, 1.62) |
| Central Sub-Saharan Africa | 115301.3 (69161.1, 172034.8) | 3.35 (3.31, 3.38) | 333.7 (200.1, 497.9) | 0.17 (0.14, 0.21) | 5363.1 (1831.7, 12116.1) | 4.43 (4.05, 4.81) | 15.5 (5.3, 35.1) | 1.23 (0.86, 1.6) |
| Eastern Sub-Saharan Africa | 406879.4 (245799.6, 613592.9) | 3.22 (3.2, 3.25) | 357.7 (216.1, 539.4) | 0.25 (0.23, 0.26) | 15899.1 (6173.1, 35205.2) | 3.23 (2.89, 3.58) | 13.9 (5.4, 30.9) | 0.25 (-0.07, 0.58) |
| Southern Sub-Saharan Africa | 91306.6 (56905.1, 139156.9) | 1.71 (1.62, 1.8) | 373.1 (232.5, 568.5) | 0.07 (0.05, 0.09) | 3110.1 (2004.1, 4860.7) | 2.33 (2.21, 2.46) | 12.7 (8.1, 19.8) | 0.68 (0.52, 0.84) |
| Western Sub-Saharan Africa | 399258.8 (241008.9, 605183.1) | 3.39 (3.35, 3.43) | 310.7 (187.5, 471.1) | 0.09 (0.04, 0.14) | 15382.2 (7454.4, 30284.6) | 3.61 (3.39, 3.84) | 11.9 (5.8, 23.5) | 0.31 (0.09, 0.53) |

**Abbreviations:** ASR, age standardized rate per/ 100,000; EAPC, estimated annual percentage change; SDI, socio-demographic index; UI, uncertainty interval; CI, confidence interval.
